# Seasonality and Paleoecology of the Late Cretaceous Multi-Taxa Vertebrate Assemblage of “Lo Hueco” (Central Eastern Spain)

**DOI:** 10.1371/journal.pone.0119968

**Published:** 2015-03-25

**Authors:** Laura Domingo, Fernando Barroso-Barcenilla, Oscar Cambra-Moo

**Affiliations:** 1 Departamento de Geología Sedimentaria y Cambio Medioambiental, Instituto de Geociencias IGEO-CSIC-UCM, Madrid, Spain; 2 Departamento de Paleontología, Universidad Complutense de Madrid, Madrid, Spain; 3 Earth and Planetary Sciences Department, University of California Santa Cruz, Santa Cruz, California, United States of America; 4 Grupo de Investigación IberCreta, Departamento de Geología y Geografía, Universidad de Alcalá de Henares, Alcalá de Henares, Spain; 5 Laboratorio de Poblaciones del Pasado, Departamento de Biología, Facultad de Ciencias, Universidad Autónoma de Madrid, Madrid, Spain; NYIT College of Osteopathic Medicine, UNITED STATES

## Abstract

Isotopic studies of multi-taxa terrestrial vertebrate assemblages allow determination of paleoclimatic and paleoecological aspects on account of the different information supplied by each taxon. The late Campanian-early Maastrichtian “Lo Hueco” Fossil-Lagerstätte (central eastern Spain), located at a subtropical paleolatitude of ~31°N, constitutes an ideal setting to carry out this task due to its abundant and diverse vertebrate assemblage. Local δ^18^O_PO4_ values estimated from δ^18^O_PO4_ values of theropods, sauropods, crocodyliforms, and turtles are close to δ^18^O_H2O_ values observed at modern subtropical latitudes. Theropod δ^18^O_H2O_ values are lower than those shown by crocodyliforms and turtles, indicating that terrestrial endothermic taxa record δ^18^O_H2O_ values throughout the year, whereas semiaquatic ectothermic taxa δ^18^O_H2O_ values represent local meteoric waters over a shorter time period when conditions are favorable for bioapatite synthesis (warm season). Temperatures calculated by combining theropod, crocodyliform, and turtle δ^18^O_H2O_ values and gar δ^18^O_PO4_ have enabled us to estimate seasonal variability as the difference between mean annual temperature (MAT, yielded by theropods) and temperature of the warmest months (TWMs, provided by crocodyliforms and turtles). ΔTWMs-MAT value does not point to a significantly different seasonal thermal variability when compared to modern coastal subtropical meteorological stations and Late Cretaceous rudists from eastern Tethys. Bioapatite and bulk organic matter δ^13^C values point to a C_3_ environment in the “Lo Hueco” area. The estimated fractionation between sauropod enamel and diet is ~15‰. While waiting for paleoecological information yielded by the ongoing morphological study of the “Lo Hueco” crocodyliforms, δ^13^C and δ^18^O_CO3_ results point to incorporation of food items with brackish influence, but preferential ingestion of freshwater. “Lo Hueco” turtles showed the lowest δ^13^C and δ^18^O_CO3_ values of the vertebrate assemblage, likely indicating a diet based on a mixture of aquatic and terrestrial C_3_ vegetation and/or invertebrates and ingestion of freshwater.

## Introduction

The Mid-Cretaceous thermal maximum, which peaked in Turonian times and constituted the warmest climate warming of the last 144 Ma [1–2] was followed by a long-term cooling trend beginning at the early Campanian and detected in both the terrestrial and marine realms [3–8]. This trend was characterized by alternating cooling and warming episodes across the Campanian and Maastrichtian [9–11]. In spite of this cooling pattern, overall warmer conditions than today persisted until the end of the Cretaceous, as suggested by high atmospheric CO_2_ concentrations and a lack of permanent ice at the poles [11–14]. During this time, enhanced ocean heat transport along with the maintainance of a low albedo at high latitudes, due to the presence of forests, contributed to a reduced latitudinal thermal gradient [15].

Although stable isotope studies on Cretaceous terrestrial vertebrate taxa can help to better understand continental climatic and environmental conditions, they are not as common as those performed on Cenozoic vertebrate taxa due to the scarcity of adequate sample sizes of fossil material, the destructive nature of the stable isotope technique, as well as pervasive diagenetic effects observed in fossils of this age. Some stable isotope studies on Cretaceous vertebrate assemblages have focused on the analysis of δ^18^O on the bioapatite with the aim of characterizing paleoclimate [16–18], faunal behavior and/or paleohydrology [18–20], and metabolism with special interest on dinosaurian body temperature [21–23]. δ^13^C values have been used to make inferences on paleoecology and to study the fractionation between diet (vegetation) and dinosaur enamel [24–26]. Domingo *et al*. [27] carried out a preliminary stable isotope study on the “Lo Hueco” vertebrate paleofauna (central eastern Spain) as a first approach to evaluate the potential, validity and degree of preservation of the bioapatite from this locality and as a first approach to characterize paleoclimatic (δ^18^O_H2O_, temperature) and paleoecological/paleoenvironmental (diet, type of vegetation) variables existing during the late Campanian-early Maastrichtian in the southeastern area of the Iberian region. Albeit the Late Cretaceous Iberian geological record shows a good representation of vertebrate localities and outcrops, there are very scarce stable isotope studies on this area and time period [27–28]. Therefore, the isotopic investigation of the “Lo Hueco” vertebrate assemblage provide valuable data, which may shed light on climatic and environmental conditions that occurred in this subtropical setting located in the Tethys realm.

The main objectives of our study are three-fold: 1) to determine seasonal thermal variability in the Iberian Late Cretaceous, 2) to characterize the isotopic offset between δ^13^C values of dinosaur tooth enamel and their diet (Δ^13^C_enamel-diet_), and 3) to unveil dietary and habitat affinities for “Lo Hueco” crocodyliforms and turtles. The richness and diversity of the Late Cretaceous “Lo Hueco” Fossil-Lagerstätte [29–30] have allowed us to approach the aforementioned questions by performing multi-taxa stable isotope analyses (δ^18^O_PO4_, δ^18^O_CO3_ and δ^13^C) on dinosaurs, crocodyliforms, turtles, gars and bulk organic matter. Most isotopic studies dealing with Mesozoic vertebrates focused on one or two groups [16–17, 19, 25–26, 31–32], so the advantage of considering co-existing taxa relies on the fact that they can shed light on differences in habitat and resource utilization, informing about climatic, environmental and ecological variables such as hydrology of the area, seasonality, type of vegetation, resource/habitat overlap and/or partitioning.

While different paleontological, geochemical and modelling studies offered a good characterization of long-term climatic variability for the Middle and Late Cretaceous (e.g., [1–3, 6, 16, 33], among others), the evaluation of seasonal thermal variability has proven challenging on account of the lack of proxies capable of recording intra-annual information. Controversy arises when discerning whether Middle and Late Cretaceous climate was more seasonally thermally equable (low mean annual range of temperatures) [34–39], although, there seems to be an agreement indicating a higher seasonal thermal variability (i.e., lower equability) during the colder Late Cretaceous in comparison to the warmer Middle Cretaceous [38].

δ^18^O values in the phosphate and carbonate fractions of vertebrate bioapatite record the δ^18^O value of their body water (δ^18^O_bw_). In the case of terrestrial endothermic species, δ^18^O_bw_ is a reflection of oxygen uptake (inspired O_2_ and water vapor, drinking water, dietary water, oxygen in food dry matter) and loss (excreted water and solids, expired CO_2_, and water vapor). Dinosaur metabolism is a controversial issue that remains under debate [21, 40–45]. Fricke and Rogers [22] performed δ^18^O_PO4_ analyses on Late Cretaceous theropods and crocodilians from fossil sites located at different latitudes and found that the reconstructed theropod slope of the δ^18^O_PO4_-latitude relationship was steeper than the one observed for crocodilians (ectotherms) and similar to that estimated for present-day endotherms such as mammals and birds. Amiot *et al*. [23] used a similar approach to that of Fricke and Rogers [22] on a wider suite of Cretaceous dinosaurs and obtained body temperatures of 36–38°C for dinosaurs inhabiting high and low latitudes. Other authors such as O’Connor and Dodson [46] and Gillooly *et al*. [47] argued that dinosaur body temperature depended on body mass (inertial homeothermy), ranging from 25°C at 12 kg to 41°C at 13,000 kg [47]. However, Fricke and Rogers [22] observed similar metabolisms for Late Cretaceous theropods showing different body mass (e.g., *Albertosaurus*: 1.3–1.7 tons; *Majungasaurus*: 1.5 tons; *Saurornitholestes*: 10 kg). Amiot *et al*. [23] observed a widespread endothermy in different groups of Late Cretaceous dinosaurs (theropods, sauropods, ornithopods, and ceratopsians). Eagle *et al*. [48] performed clumped isotope analyses on sauropods and estimated body temperatures between 4 and 7°C lower (36 to 38°C) than those proposed by Gillooly *et al*. [47], arguing that sauropods were able to regulate their body temperatures, preventing overheating. Ectothermic semi-aquatic taxa, such as crocodiles and turtles, form their bioapatite within a narrow thermal window, and their bioapatite δ^18^O is a reflection of the δ^18^O_H2O_ value of warm months when conditions are favorable for apatite synthesis [20, 49–50]. In the case of ectothermic aquatic taxa (gars), bioapatite forms in isotopic equilibrium with ambient water and since they do not thermoregulate, their bioapatite δ^18^O values record both δ^18^O_H2O_ and ambient temperature independently of body temperature [51]. Therefore, it is possible to estimate temperature values from gar δ^18^O values if the δ^18^O_H2O_ value is independently estimated [20, 51–52].

Studies dealing with dinosaur δ^13^C values have mainly focused on herbivore taxa (e.g., [24–26, 53]). These authors obtained relatively high δ^13^C values for hadrosaurian and ceratopsian dinosaurs for a typical C_3_ environment (C_4_ plant expansion took place in the late Miocene–Pliocene). Plants following the C_3_ or Calvin-Benson photosynthetic pathway (trees, shrubs, forbs, and cool-season grasses) strongly discriminate against ^13^C during fixation of CO_2_, yielding tissues with δ^13^C values averaging ∼ -27.5‰ (VPDB) (ranging from -36‰ to -22‰). The most negative δ^13^C values of this range (-36‰ to -30‰) reflect closed canopy conditions due to recycling of ^13^C-depleted CO_2_ and low irradiance. The highest values (-25‰ to -22‰) correspond to C_3_ plants from high insolated, arid, or water stressed environments [54–56]. When considering fossil taxa, it is necessary to account for shifts in the δ^13^C value of atmospheric CO_2_ (δ^13^C_atmCO2_), the source of plant carbon, including anthropogenic modification due to fossil fuel burning, which has decreased the δ^13^C value of atmospheric CO_2_ from a value of −6.0‰ in the Late Cretaceous [57] to a modern value of −8.0‰ [58–59]. Accounting for these shifts in baseline and assuming the modern fractionation of ∼ -19.5‰ between δ^13^C_atmCO2_ and C_3_ vegetation δ^13^C values [60], Late Cretaceous mean δ^13^C value for C_3_ plants would be ∼ -25.5‰ (ranging from -34‰ to -20‰), with the most negative δ^13^C values (-34‰ to -28‰) reflecting closed canopy conditions and the highest values (-23‰ to -20‰) reflecting high insolated, arid, or water stressed environments. Studies of δ^13^C offsets between terrestrial vertebrate enamel and diet (Δ^13^C_enamel-diet_) have been mainly carried out on modern mammalian taxa, which allows for extrapolation on paleontological studies dealing with mammals, so that a consistent Δ^13^C_enamel-diet_ of +12 to +14‰ has been observed between herbivore mammalian tooth enamel and vegetation δ^13^C values [61–63], while the difference between mammalian carnivore and herbivore tooth enamel δ^13^C values (Δ^13^C_carnivore-herbivore_) has been proposed to be ∼ -1.3‰ [64]. Johnson *et al*. [65] and Angst *et al*. [66] also investigated this tissue-diet fractionation on modern birds by analyzing ostrich eggshells and diet, obtaining a Δ^13^C_eggshell-diet_ value of +16‰ and +13.4‰, respectively. The lack of modern counterparts in the case of dinosaurs makes it difficult to assess this offset and we can just rely on the fossil record geochemical imprint to determine tissue-diet fractionation values. In this vein, analyzing herbivore dinosaur bioapatite and bulk organic matter δ^13^C values, Fricke and Pearson [25] and Fricke *et al*. [26] argued that Δδ^13^C_enamel-diet_ for ornithischian dinosaurs was ∼ +18‰, whereas Tütken [32] estimated a Δδ^13^C_enamel-diet_ value of ∼ +16‰ for sauropods.

### Geographic and geological setting

**Fig 1:**
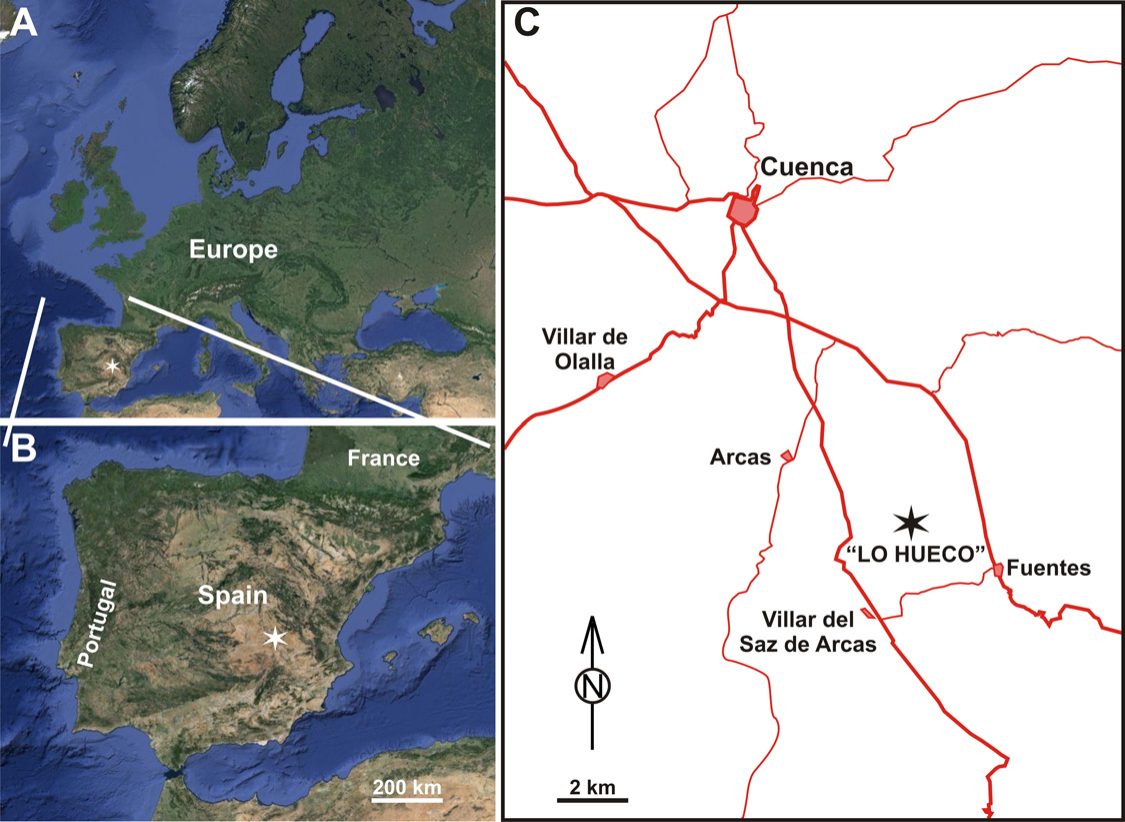
Geographic setting of the “Lo Hueco” fossil site. Satellite images of A) Europe, and B) Iberian Peninsula; the star shows the situation of the “Lo Hueco” fossil site. C) Close-up view of “Lo Hueco” near the town of Fuentes showing other towns, and the main and secondary roads. Europe and Iberian Peninsula satellite images from USGS EROS (Earth Resources Observatory and Science (EROS) Center) http://eros.usgs.gov/#.

**Fig 2:**
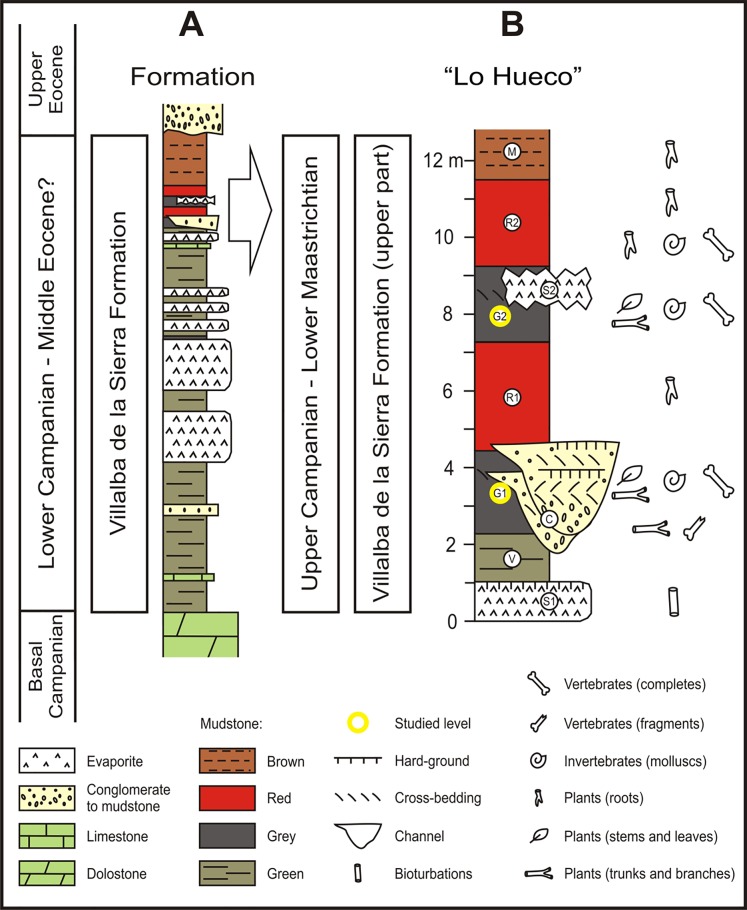
Stratigraphic context of the “Lo Hueco” fossil site. A) The Villalba de la Sierra Formation. B) Detailed stratigraphic section of “Lo Hueco” showing the studied levels G1 and G2.

The Late Cretaceous “Lo Hueco” Fossil-Lagerstätte is located in the province of Cuenca (central eastern Spain: 2° 02’50”W, 39° 57’15”N) (Fig. 1). It was fortuitously discovered in 2007, while constructing the Madrid-Levante high-speed railway. More than 10,000 macrofossils of different taxonomic groups of flora and fauna were unearthed. The stratigraphic position and associated fauna support a late Campanian–early Maastrichtian age [29, 67–68]. The “Lo Hueco” site outcrops in Garumn facies, which is the informal term that designates marl, clay, and gypsum, mainly of red color, deposited in shallow marine, coastal, and/or continental environments of southwestern Europe during the latest Cretaceous and the earliest Paleogene [69]. It corresponds to the upper part of the Villalba de la Sierra Formation (Fig. 2A), a lithological unit interpreted as a coastal marsh with distributary channels and sporadic establishment of sabkhas [29]. Six stratigraphic levels were defined at the “Lo Hueco” outcrop named V, G1, R1, G2, R2, and M from bottom to top [29] (Fig. 2B). This succession appears slightly modified laterally by a lower sulphate interval (S1) that cuts the V level in the eastern area of the outcrop, by a sandy channel structure (C) that interrupts V, G1, and R1 levels in the southern area, and by an upper sulphate interval (S2) that cuts part of G2 in the northeastern area (Fig. 2B). Four bonebeds have yielded the majority of fossils: the C structure, the G1 and G2 levels and the lower part of the R2 level (Fig. 2B) [29, 68]. The G1 level is interpreted as a proximal muddy floodplain (close to the distributary channels) and vertebrate fossils usually appear complete and associated. More than 14 partially articulated sauropod skeletons were recovered from this level [28, 68]. Exceptionally well-preserved plant remains have been also described from this lithosome [30]. The G2 level corresponds to the distal part of a poorly drained muddy floodplain (distal from the distributary channels) and points to a relatively calm aquatic environment (i.e., marsh/swamp) exposed to partial or total desiccation, although there is a low degree of articulation of preserved skeletons [29, 68]. Recent fluid inclusion and geochemical analyses on the sulphates from S1 and S2 have pointed to a near coast playa-lake environment with mainly brackish and freshwater influence in the area, while not totally ruling out some extent of marine influence [70]. During the late Campanian-early Maastrichtian, the “Lo Hueco” locality was placed at a subtropical paleolatitude of ∼ 31°N.

## Materials and Methods

The rich and diverse vertebrate assemblage of the “Lo Hueco” fossil site allowed us to collect multi-taxa samples including dinosaur (theropods: n = 11, sauropods: n = 4) and crocodyliform (n = 36) tooth enamel, turtle shell (n = 9) and gar ganoine (n = 19) from levels G1 and G2. Paleontological prospection and excavation undertaken at the “Lo Hueco” fossil site were authorized by the Dirección General de Patrimonio y Museos of the Junta de Comunidades de Castilla-La Mancha (Spain) (permit number: 04–0392-P11). “Lo Hueco” vertebrate remains are housed at Museo de las Ciencias de Castilla-La Mancha (Cuenca, Spain). Complete repository information including specimen number, stratigraphic situation and geographic location are given in S1 Dataset. Dinosaurs, crocodyliforms and turtles were analyzed for their δ^18^O_PO4_, δ^18^O_CO3_ and δ^13^C values, whereas gars were analyzed for their δ^18^O_PO4_ values. Dinosaur dentine was also analyzed in order to check for diagenetic effects. Although three major groups of dinosaurs (theropods, sauropods and ornithopods) are represented in “Lo Hueco”, in this study we had access to theropods belonging to Dromaeosaurinae and Velociraptorinae Eumaniraptora and to sauropods, which are presumably representing two titanosaurian species, one of them close to the genus *Ampelosaurus* [71–73]. Eusuchian crocodile remains are very abundant at this locality, although the record is monotonous and seems to be represented by two genera close to *Allodaposuchus* [74]. Most of the turtle material probably belongs to two forms of Pleurodira, specifically to members of Bothremydidae [29, 75–76]. Finally, actinopterygians from “Lo Hueco” are mainly represented by lepisosteids, commonly known as gars [29]. We also performed δ^13^C analyses on bulk organic matter from the “Lo Hueco” G1 (n = 10) and G2 (n = 8) levels with the aim of characterizing the type of vegetation that was present in the area.

The carbon and oxygen isotope results are reported in the δ-notation. δ^H^X_sample_ = [(R_sample_—R_standard_)/R_standard_] × 1000, where X is the element, H is the mass of the rare, heavy isotope, and R = ^13^C/^12^C or ^18^O/^16^O. Vienna Pee Dee Belemnite (VPDB) is the standard for δ^13^C values, whereas δ^18^O values for both bioapatite carbonate and phosphate are reported relative to Vienna Standard Mean Ocean Water (VSMOW).

Sample chemical treatments followed those described in Bassett *et al*. [77] for phosphate in bioapatite, Koch *et al*. [78] for carbonate in bioapatite and Domingo *et al*. [79] for bulk organic matter. All samples were analyzed at the Stable Isotope Laboratory of the University of California Santa Cruz (USA). Bioapatite δ^18^O_PO4_ values were measured using a Thermo Finnigan Delta plus XP isotope ratio mass spectrometer coupled via continuous flow to a high temperature conversion elemental analyzer (TCEA). Bioapatite δ^13^C and δ^18^O_CO3_ analyses were conducted on a Thermo MAT253 dual-inlet isotope-ratio mass spectrometer coupled to a Kiel IV carbonate device. Bulk organic matter δ^13^C analyses were performed using a Carlo Erba 1108 elemental analyzer interfaced to a ThermoFinningan Delta Plus XP isotope ratio mass spectrometer. The standards used in the case of phosphates were Fisher Standard (δ^18^O = 8.4‰), UCSC Low standard (δ^18^O = 11.3‰), UCSC High standard (δ^18^O = 19.0‰) and Kodak standard (δ^18^O = 18.1‰) (all of them are silver phosphate). Standard deviations for repeated measurements of Fisher (n = 52), UCSC Low (n = 13), UCSC High (n = 16), and Kodak (n = 7) standards were 0.51‰, 0.55‰, 0.21‰, and 0.36‰ for δ^18^O_PO4_. As suggested by Suarez *et al*. [20], we used NIST 120c as a quality control standard and not as a calibration standard since its value is highly dependent on both pretreatment and analysis methods. NIST 120c value (n = 8) was 21.5±0.4. Replicate δ^18^O_PO4_ analyses were carried out in ∼80% of the samples. The average absolute difference for δ^18^O_PO4_ was 0.02‰ and the standard deviation of this average difference was 0.20‰. The standards used in the case of carbonates were Carrara Marble (CM, δ^13^C = 2.05‰ and δ^18^O = -1.91‰, both VPDB), NBS-18 (δ^13^C = -5.03‰ and δ^18^O = -23.01‰, VPDB) and NBS-19 (δ^13^C = 1.95‰ and δ^18^O = -2.20‰, VPDB). The standard deviations for repeated measurements of CM (n = 16), NBS-18 (n = 19), and NBS-19 (n = 10) were 0.03‰, 0.33‰, and 0.04‰ for δ^13^C, respectively, and 0.08‰, 0.07‰, and 0.09‰ for δ^18^O, respectively. Duplicate analyses were performed for ∼50% of the samples. The average absolute difference for δ^13^C and δ^18^O_CO3_ was 0.03‰ and 0.14‰, respectively, and the standard deviations of these average differences were 0.02‰ and 0.05‰ for δ^13^C and δ^18^O_CO3_, respectively. The standards used in the case of bulk organic matter analyses were Pugel (δ^13^C = -12.60‰) and Acetanilide (δ^13^C = -29.53‰). The standard deviations for repeated measurements of Pugel (n = 11) and Acetanilide (n = 3) were 0.15‰ and 0.02‰, respectively. Statistical tests were performed using the program SPSS PASW Statistics 18.0 software.

We used the following equations to calculate δ^18^O_H2O_ values from “Lo Hueco” vertebrates:

Dinosaurs: δ^18^O_H2O_ = 1.11δ^18^O_PO4_–26.44 [16] (1)

Crocodyliforms: δ^18^O_H2O_ = 0.82δ^18^O_PO4_–19.13 [80] (2)

Turtles: δ^18^O_H2O_ = 1.06δ^18^O_PO4_–21.6 [81] (3)

Equation (1) was calculated by Amiot *et al*. [16] constructing a database with modern mammalian δ^18^O_PO4_ values and the δ^18^O value of meteoric water from IAEA stations. Since most of dinosaur samples from “Lo Hueco” belong to theropods, we considered them as endotherms following most authors [22–23, 45], and therefore we applied equation (1) as Amiot *et al*. [16] suggested in their study. Equation (2) was experimentally determined by Amiot *et al*. [80] analyzing modern crocodilian δ^18^O_PO4_ values and the δ^18^O value of the water in which they live. Equation (3) was determined by Coulson *et al*. [81] for freshwater and marine extant turtle datasets combining experimental and literature results. This equation was adjusted to correct for the difference in NIST 120c value obtained by Coulson *et al*. [81] (22.6‰) and in our study (21.5±0.4).

Once dinosaur, crocodyliform and turtle δ^18^O_H2O_ values were estimated, we used gar ganoine δ^18^O_PO4_ values as an independent proxy to calculate temperature values and to eventually determine temperature offsets related to seasonal patterns (see Discussion). Following Domingo *et al*. [82], we selected lepisosteid middle flank scales since they record the greatest number of layers of ganoine per unit of time and grow all year round. In the case of dinosaurs, we have only used theropod δ^18^O_PO4_ values on account of 1) the low number of sauropod samples, and 2) the uncertainty about whether sauropods obtained water mainly from drinking (i.e., obligate drinkers) or from vegetation (i.e., non-obligate drinkers).

Different phosphate-water oxygen isotope fractionation equations have been proposed as paleothermometers based mainly on studies carried out on extinct and extant fish and invertebrate phosphates. The utilization of a given equation may yield significant differences in calculated temperature values when compared to other equations, which can be as large as 8–9°C [83–84]. Here, we applied three different equations in order to check whether they can be used to calculate temperature offsets related to seasonal patterns independently of the absolute temperature values yielded by each of them (see Discussion). We considered the following equations:

T(°C) = 119.3 (±12.9)– 4.38 (±0.54) (δ^18^O_PO4_ –δ^18^O_H2O_) [51] (4)

This equation was rescaled for a NBS 120b value of 21.4‰ (see [84])

T(°C) = 118.7 (±4.9)– 4.22 (±0.20) (δ^18^O_PO4_ –δ^18^O_H2O_) [85] (5)

T(°C) = 117.4 (±9.5)– 4.50 (±0.43) (δ^18^O_PO4_ –δ^18^O_H2O_) [84] (6)

## Results

Table 1 shows dinosaur, crocodyliform, turtle and gar mean δ^18^O_PO4_, δ^13^C, δ^18^O_CO3_, and δ^18^O_H2O_ values. Individual values per sample and statistical tests are given in S1 and S2 Datasets, respectively.

**Table 1:** Mean and standard deviation (SD) δ^18^O_PO4_ (‰ VSMOW), δ^18^O_H2O_ (‰ VSMOW), δ^13^C (‰ VPDB), and δ^18^O_CO3_ (‰ VSMOW) values of “Lo Hueco” theropods, sauropods, crocodyliforms, turtles and gars for the total dataset (TOTAL) and independently for G1 and G2 levels.

	δ^18^O_PO4_ (‰ VSMOW)		δ^18^O_H2O_ (‰ VSMOW)		δ^13^C (‰ VPDB)		δ^18^O_CO3_ (‰ VSMOW)	
	Mean	SD	Mean	SD	Mean	SD	Mean	SD
**TOTAL**								
**Theropods**	20,8	0,9	-3,5	1,0	-10,7	0,8	30,0	1,1
**Sauropods**	20,9	0,4	-	-	-10,5	0,8	29,0	0,1
**Crocodyliforms**	19,4	0,9	-3,2	0,7	-10,4	1,9	28,2	1,0
**Turtles**	18,3	0,7	-2,2	0,7	-11,8	0,6	27,1	0,3
**Gars**	19,8	1,0						
**G1**								
**Theropods**	20,2	1,2	-4,1	1,4	-10,7	1,2	31,2	0,2
**Sauropods**	20,6	-	-	-	-10,6	1,0	29,0	0,02
**Crocodyliforms**	19,0	0,7	-3,6	0,6	-10,3	2,1	28,1	1,1
**Turtles**	17,9	0,6	-2,7	0,6	-12,5	0,1	27,0	0,3
**Gars**	19,1	1,5						
**G2**								
**Theropods**	20,7	0,5	-3,5	0,5	-10,9	0,6	29,7	1,1
**Sauropods**	21,1	-	-	-	-10,3	0,9	29,0	0,1
**Crocodyliforms**	20,0	0,7	-2,7	0,6	-10,6	1,8	28,2	1,1
**Turtles**	18,7	0,5	-1,8	0,5	-11,3	0,2	27,2	0,2
**Gars**	19,7	0,9	-	-	-	-	-	-

### δ^18^O_PO4_ results

**Fig 3:**
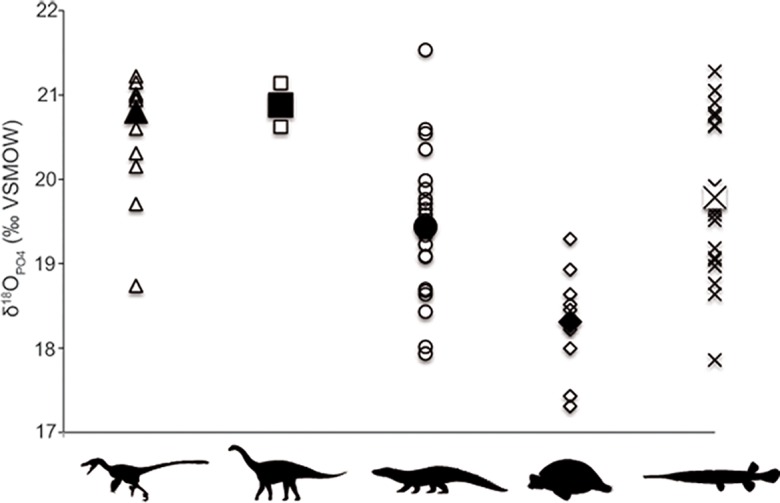
δ^18^O_PO4_ (‰ VSMOW) values of “Lo Hueco” vertebrates. Theropods (triangles), sauropods (squares), crocodyliforms (circles), turtles (diamonds), and gars (crosses). Large black symbols represent mean δ^18^O_PO4_ values.

Fig. 3 shows mean δ^18^O_PO4_ values for theropods, sauropods, crocodyliforms, turtles and gars. Mean δ^18^O_PO4_ value for theropods is 20.8±0.9‰, with a non-significant increase of ∼0.5‰ between G1 and G2 (t = -1.146, p = 0.276). Mean δ^18^O_PO4_ value for sauropods is 20.9 ± 0.4‰ and there is also an increase of ∼0.5‰ in δ^18^O_PO4_ between G1 and G2, although in this case no statistical tests were done since only one sauropod per level could be sampled. Mean δ^18^O_PO4_ value for crocodyliforms is 19.4 ± 0.9‰ with a significant increase of ∼1.0‰ between G1 and G2 (t = -3.491, p = 0.002). Turtles show a mean δ^18^O_PO4_ value of 18.3‰ ± 0.7‰, with a non-significant increase of ∼0.8‰ between G1 and G2 (t = -2.208, p = 0.063). Crocodyliforms and turtles show statistically consistent lower δ^18^O_PO4_ values than dinosaurs for the total dataset (Table 1 and S2 Dataset). Mean δ^18^O_PO4_ value for gars is 19.8 ± 1.0‰, with a non-significant decrease of ∼0.2‰ between G1 and G2 (t = 0.475, p = 0.641). ANOVA tests show significant differences for δ^18^O_PO4_ values when comparing all taxa for the total dataset and for G1 and G2 independently (S2 Dataset).

### δ^18^O_H2O_ results

Mean calculated δ^18^O_H2O_ value for theropods is -3.5 ± 1.0‰, with a non-significant increase of ∼0.6‰ between G1 and G2 (t = -0.670, p = 0.566). Mean δ^18^O_H2O_ value for crocodyliforms is -3.2 ± 0.7‰, with a significant increase of ∼0.9‰ between G1 and G2 (t = -2.541, p = 0.019). Turtle mean δ^18^O_H2O_ value is -2.2 ± 0.7‰, with a non-significant increase of ∼0.9‰ between G1 and G2 (t = -2.215, p = 0.062). ANOVA test perfomed for the total dataset and for levels G1 and G2 showed significant differences in δ^18^O_H2O_ value among theropods, crocodyliforms and turtles (S2 Dataset).

### δ^13^C and δ^18^O_CO3_ results

**Fig 4:**
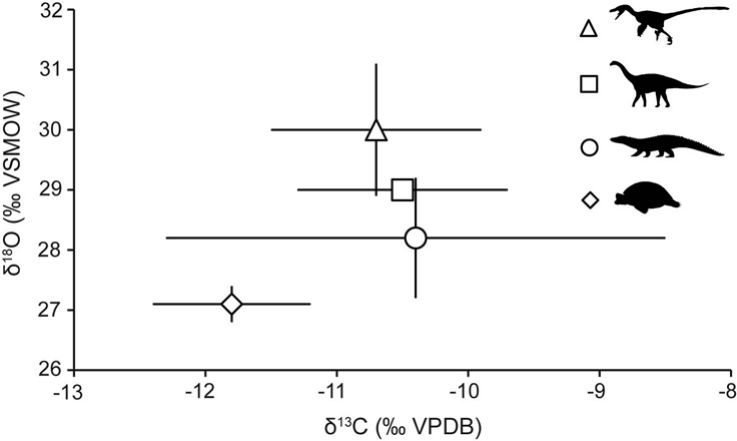
Mean ± 1 SD δ^13^C (‰ VPDB) and δ^18^O_CO3_ (‰ VSMOW) values of the “Lo Hueco” vertebrates. Theropods (triangles), sauropods (squares), crocodyliforms (circles), and turtles (diamonds).

Fig. 4 shows a biplot δ^13^C- δ^18^O_CO3_ graph for the “Lo Hueco” vertebrate assemblage. Theropod mean δ^13^C and δ^18^O_CO3_ values are -10.7 ± 0.8‰ and 30.0 ± 1.1‰, respectively, with a decrease in both values between G1 and G2, although only significant in the case of δ^18^O_CO3_ (t = 2.603, p = 0.022). Sauropod mean δ^13^C and δ^18^O_CO3_ values are -10.5 ± 0.8‰ and 29.0 ± 0.1‰, respectively, with a non-significant increase in δ^13^C between G1 and G2 (t = -0.334, p = 0.770) and with the same δ^18^O_CO3_ value in both levels. Crocodyliform mean δ^13^C and δ^18^O_CO3_ values are -10.4 ± 1.9‰ and 28.2 ± 1.0‰, respectively, with a non-significant decrease in δ^13^C between G1 and G2 (t = 0.214, p = 0.834) and a non-significant increase in δ^18^O_CO3_ between G1 and G2 (t = -0.204, p = 0.842). Finally, turtle mean δ^13^C and δ^18^O_CO3_ values are -11.8 ± 0.6‰ and 27.1 ± 0.3‰, respectively, with increases in both values between G1 and G2, although only significant in the case of δ^13^C (t = -14.300, p <0.001). δ^13^C values do not show statistically significant differences among taxa for the total dataset, G1 and G2, whereas δ^18^O_CO3_ values do show significant differences in all cases (S2 Dataset).

Bulk organic matter mean δ^13^C value is -25.1±1.4‰, with a non-significant increase of ∼0.2‰ between G1 and G2 (t = -0.302, p = 0.767).

### Diagenesis

**Fig 5:**
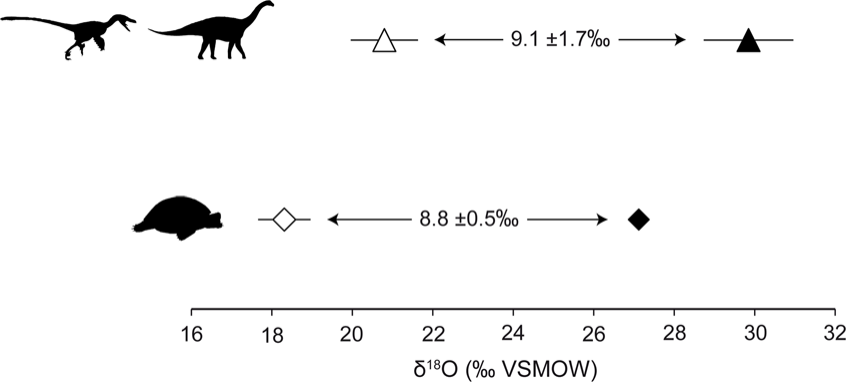
Δ^18^O_CO3-PO4_ values for dinosaurs (theropods and sauropods) and turtles. Both values are within the range of the expected equilibrium difference for modern bioapatites (∼8.6–9.1‰) [87–88]. Open symbols represent δ^18^O_PO4_ values and solid symbols correspond to δ^18^O_CO3_.

Several lines of evidence point to a low degree of diagenetic alteration for the “Lo Hueco” fossil vertebrates:

1) Tooth enamel was the selected tissue for dinosaurs, crocodyliforms, whereas ganoine was analyzed in the case of gars. Relative to dentine, bone and isopedine, tooth enamel and ganoine have larger apatite crystals, a lower content of organic matter, and a low porosity, making them the least prone tissues to undergo diagenetic alteration [86].

2) Several studies of living organisms point to a difference between bioapatite δ^18^O_CO3_ and δ^18^O_PO4_ values (Δ^18^O_CO3-PO4_) of ∼8.6–9.1‰ [87–88]. Obtaining a Δδ^18^O_CO3-PO4_ value near this range in fossil bioapatite has been viewed as an indication that both phases retain pristine isotopic values [87, 89–91]. The mean Δ^18^O_CO3-PO4_ value for “Lo Hueco” dinosaurs is 9.1 ± 1.7‰ and thus, it is in the range of the expected equilibrium difference supporting a low degree of diagenetic alteration (Fig. 5). Although this relationship was established for endotherms, “Lo Hueco” turtle Δ^18^O_CO3-PO4_ value is also in that range (8.8 ± 0.5‰) (Fig. 5), probably reflecting sustained and constant body temperature during mineral growth (preferentially during summer months: [49–50]). The fact that we only sampled compact bone from the outermost part of turtle shells reduces the possibility of diagenetic alteration. Δ^18^O_CO3-PO4_ value could not be calculated for crocodyliforms since δ^18^O_CO3_ and δ^18^O_PO4_ values did not come from the same samples.

3) Dinosaur enamel and dentine δ^18^O_PO4_ analyses were performed in order to evaluate the response of these tissues against postburial alteration. Several authors have observed higher isotopic values in dinosaur dentine compared to enamel (e.g., [24–26]). Fricke and Pearson [25] argued that this difference was due to a variable exposure of dentine to diagenetic fluids, as well as more isotopic exchange and/or secondary mineral formation in dentine compared to enamel. In the case of the “Lo Hueco” dinosaurs, we also observed higher isotopic values in dentine with theropods showing Δ^18^O_PO4dentine-enamel_ = 1.04, and sauropods showing Δ^18^O_PO4dentine-enamel_ = 1.34. Significant differences have been detected between both tissues when considering the total dataset (t = -2.983, p = 0.007).

4) An additional line of evidence to assess the preservation of original δ^18^O_PO4_ values was outlined by Fricke and Rogers [22] when trying to determine the type of metabolism of dinosaurs. According to these authors, differences in body temperatures between ectotherms and endotherms give rise to differences in δ^18^O_PO4_ values as a function of latitude in such a way that at high latitudes, ectotherms have higher δ^18^O_PO4_ values than endotherms (due to lower body temperature for ectotherms). Conversely, at low latitudes, endotherms show higher δ^18^O_PO4_ values than ectotherms. Amiot *et al*. [16] stated that diagenetic processes would bring about the homogenization of isotopic values and therefore, the aforementioned differences would not be observable. The “Lo Hueco” fossil site was situated at a low paleolatitude (∼31°N) during the Late Cretaceous [92]. Theropod dinosaurs, which have been proposed to be endotherms [22–23, 93], show consistently higher δ^18^O_PO4_ values in comparison to crocodyliforms and turtles (ectotherms) at the “Lo Hueco” fossil site when considering the total dataset as well as G1 and G2 levels separately (Table 1), in agreement with the results observed by Fricke and Rogers [22] and Amiot *et al*. [16]. In addition to the difference observed in δ^18^O_PO4_ values between endotherms and ectotherms, lower δ^18^O_PO4_ values for aquatic gars and semi-aquatic crocodyliforms and turtles, and higher δ^18^O_PO4_ values for terrestrial theropods constitute another line of evidence pointing to the preservation of the original isotopic signal, as previously stated by other authors [19–20].

## Discussion

### Seasonal thermal variability in the Iberian Late Cretaceous

**Fig 6:**
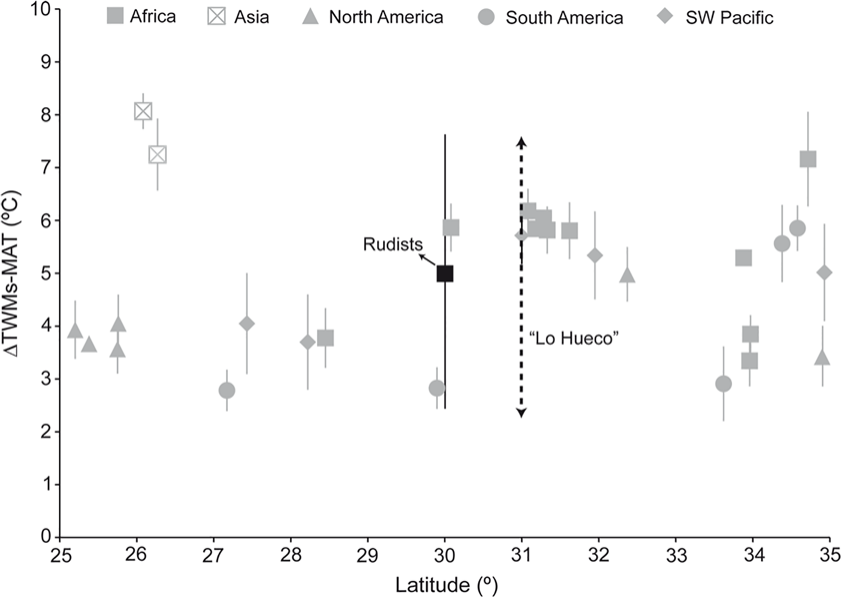
ΔTWMs-MAT (°C) *vs* latitude (°) for “Lo Hueco” (black dotted arrow), Late Cretaceous eastern Tethyan rudists from Steuber [36] and Steuber *et al*. [38] (black square), and modern meteorological stations located at latitudes 25° to 35° in both hemispheres from Africa (grey square), Asia (crossed square), North America (grey triangle), South America (grey circle), and Southwest Pacific (grey diamond). Absolute latitude is shown including North and South hemispheres.

Controversy arises when discerning whether Middle and Late Cretaceous climate witnessed a lower mean annual range of temperatures (i.e., seasonal thermal equability) than today [34–39], although it seems that during the colder Late Cretaceous there was a higher seasonal thermal variability (ie., lower equability) in comparison to the warmer Middle Cretaceous [38]. Multi-taxa studies as the one carried out here allow us to investigate this issue on account of differences in the timing of bioapatite growth, thus tracking different moments throughout the year. While in general, δ^18^O_H2O_ values calculated for theropods, crocodyliforms and turtles from “Lo Hueco” correspond to typical precipitation waters in subtropical locations [94], theropods recorded consistently lower δ^18^O_H2O_ and temperature values than crocodyliforms and turtles in G1 and G2 levels as well as in the total dataset (Table 1). Lower δ^18^O_H2O_ values of terrestrial endothermic taxa such as theropods would correspond to ingested water probably consumed during the whole year and hence, recording all seasons. In contrast, δ^18^O_H2O_ data of semiaquatic ectothermic taxa such as crocodyliforms and turtles would represent local meteoric waters over a shorter time scale when the conditions are favorable for apatite synthesis (i.e., growth during the warm season and higher δ^18^O_H2O_ values) [21, 49, 80]. Temperature values calculated from theropod δ^18^O_H2O_ values would track mean annual temperature (MAT), whereas temperature values estimated from crocodyliform and turtle δ^18^O_H2O_ values would record temperature of the warmest months (TWMs), when apatite synthesis is more likely to occur. Albeit the seasonal thermal amplitude is usually calculated as the difference between temperature of the warmest months (TWMs) *minus* temperature of the coldest months (TCMs), in our approach it is not feasible to work out this latter value with the available proxies. We opted to infer the semi-seasonal thermal variability characterized as the difference between TWMs and MAT (ΔTWMs-MAT). Since the “Lo Hueco” site was located in a coastal subtropical setting, we created a database with modern meteorological information from coastal stations situated within the subtropics in both hemispheres (25°–35°) compiling MAT and TWMs (i.e., July, August, September) data and calculating ΔTWMs-MAT (S3 Dataset). We also considered seasonal data provided by Tethyan Cretaceous rudists [36, 38]. These authors argued that during the colder Early Cretaceous (late Barremian-middle Albian), seasonal thermal variability was more intense than during warmer Cretaceous episodes (late Albian-early Campanian). Fig. 6 shows ΔTWMs-MAT *vs* subtropical latitude range (25°–35°) for data calculated from vertebrate taxa from “Lo Hueco”, modern subtropical stations, and Santonian-Campanian eastern Tethyan rudists (Greece and Turkey: [36, 38]). ΔTWMs _crocodyliforms-_MAT_theropods_ values from “Lo Hueco” are 2.2±0.1°C, 3.5±0.1°C, and 2.7±0.1°C for G1, G2, and the total dataset, respectively (S4 Dataset), whereas ΔTWMs _turtles-_MAT_theropods_ yielded higher values of 6.1±0.2°C, 7.4±0.20°C, and 6.7±0.2°C for G1, G2, and the total dataset, respectively (S4 Dataset). Similar ΔTWMs _crocodyliforms-_MAT_theropods_ and ΔTWMs _turtles-_MAT_theropods_ values were obtained when using equations (4), (5) and (6) (S4 Dataset). ΔTWMs-MAT values from modern data vary between minimum values of ∼2.8±0.4°C (Easter Island, Chile, 27.17°S) and maximum values of ∼8.1±0.3°C (Fuzhou, China, 26.08°N). Finally, the mean sea-surface ΔTWMs-MAT value from Late Cretaceous Tethyan rudists is ∼5.0±2.6°C (Greece and Turkey, ∼30°N). No significant statistical differences have been found between the “Lo Hueco” ΔTWMs-MAT values and those shown by modern meteorological stations and Cretaceous rudists (S5 Dataset) indicating that the climatic conditions in the subtropical western area of the Tethys during the late Campanian-early Maastrichtian were not more significantly equable than those observed today. Previous studies have suggested that during mid-Cretaceous greenhouse conditions the latitudinal thermal gradient was weaker than the one observed today, whereas during the cooler late-Cretaceous, the latitudinal thermal gradient has been proposed to be either weaker or similar to that observed today [6, 95–96]. Cool greenhouse periods, as Huber *et al*. [6] refer to mid-Campanian-Maastrichtian, showed warm temperatures in high latitudes and cool temperatures in the subtropics and tropics (e.g., [95]). Cooler conditions have been traditionally associated to a lower thermal equability and *vice versa* [37, 97] and in this vein, Steuber *et al*. [38], studying seasonal thermal variability on Tethyan rudists, observed a lower thermal seasonality during the warmer Cretaceous episodes, whereas cooler Cretaceous episodes show a higher thermal seasonality. “Lo Hueco” temperature data do not show a significant different seasonal thermal variability when compared to modern data pointing to a similar seasonal thermal amplitude between central eastern Iberia during the late Campanian-early Maastrichtian and today (S3 and S5 Datasets). The determination of past seasonality remains a difficult issue to determine due to the limitation of the seasonal information yielded by paleoproxies and the shortage of these indicators in terrestrial settings. Isotopic studies of multi-taxa vertebrate assemblages as the one from the “Lo Hueco” locality help to characterize past seasonality. Future clumped isotope analyses on soil carbonate and invertebrate carbonate from the “Lo Hueco” locality may allow us to double-check TWMs values and compare them with those provided by crocodyliforms and turtles.

### “Lo Hueco” vertebrate isotopic paleoecology

**Fig 7:**
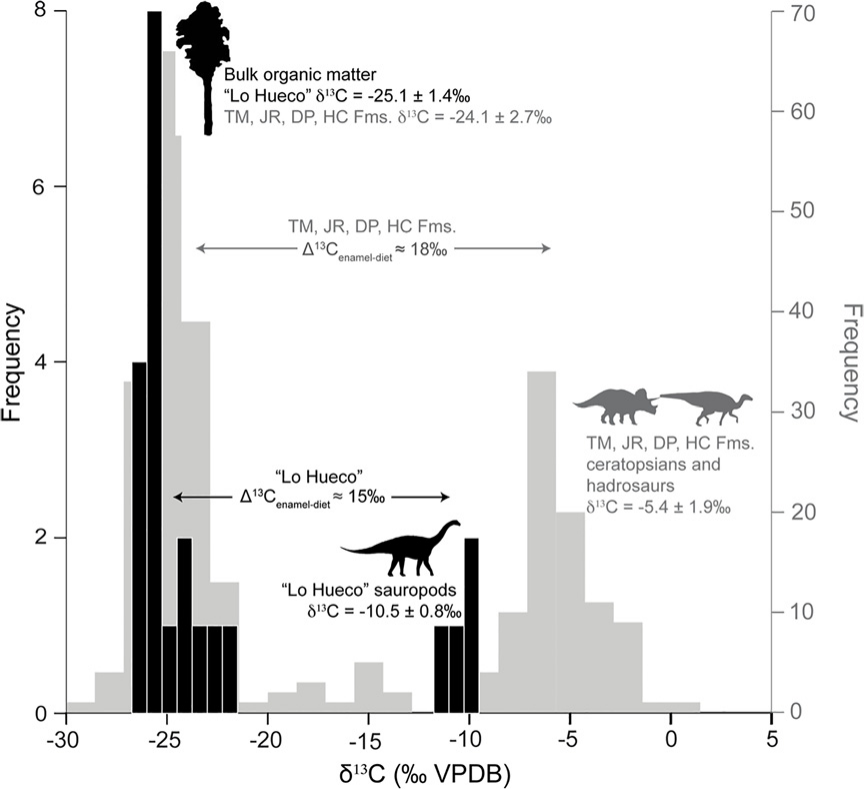
Fractionation between herbivore enamel and herbivore diet (Δ^13^C_enamel-diet_). “Lo Hueco” bulk organic matter and sauropod mean ± 1 SD δ^13^C (‰ VPDB) (black). Two Medicine (TM), Judith River (JR), Dinosaur Park (DP), and Hell Creek (HC) Formations bulk organic matter and ceratopsian and hadrosaur mean ± 1 SD δ^13^C (‰ VPDB) (grey) [25–26]. “Lo Hueco” Δ^13^C_enamel-diet_ is 15‰, when analysing sauropods, whereas Fricke and Pearson [25] and Fricke *et al*. [26] obtained a value of 18‰ when analysing ceratopsians and hadrosaurs. Note different frequency scale for “Lo Hueco” (black) and Fricke and Pearson [25] and Fricke *et al*. [26]´s data (grey).

**Fig 8:**
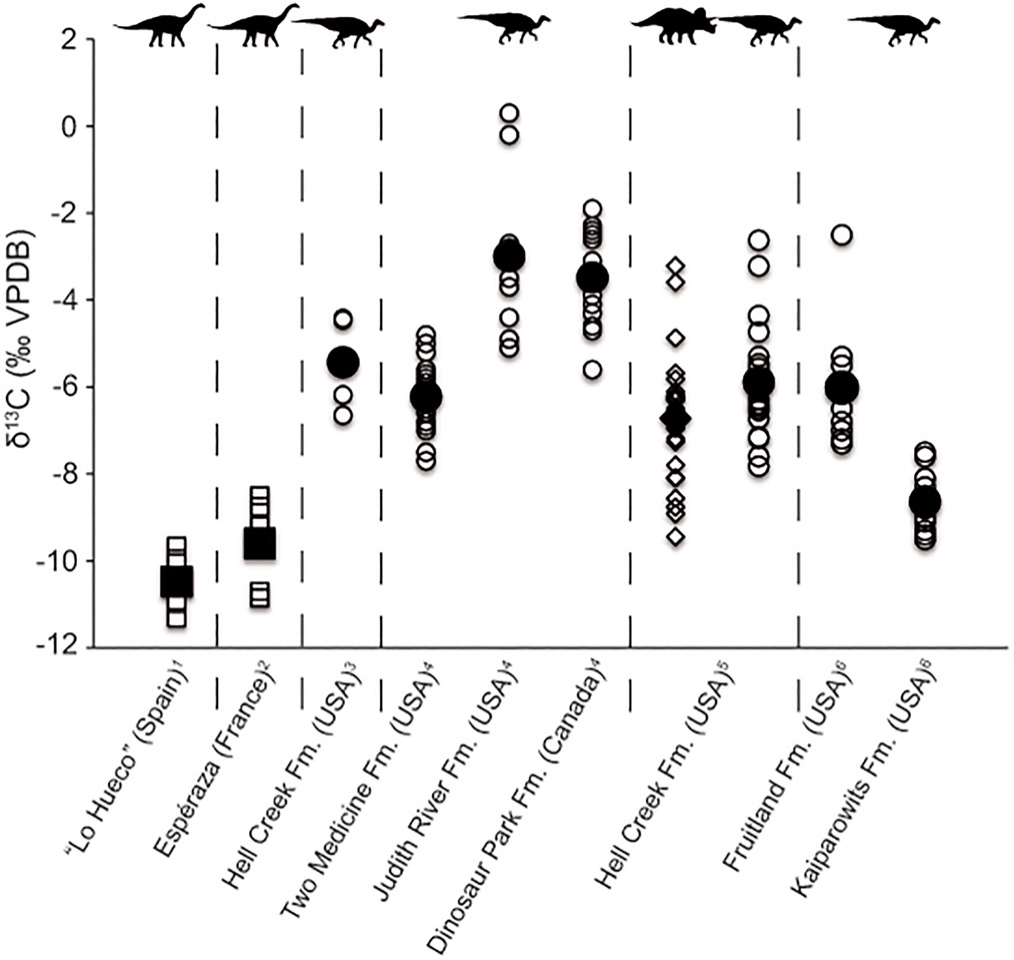
δ^13^C (‰ VPDB) values for Campanian-Maastrichtian herbivore dinosaurs: titanosaur sauropods (squares), hadrosaurs (circles), and ceratopsians (diamonds). Large black symbols represent mean δ^13^C values. ^1^This study; ^2^Tütken [32]; ^3^Stanton-Thomas and Carlson [24]; ^4^Fricke *et al*. [26]; ^5^Fricke and Pearson [25]; ^6^Fricke *et al*. [53].

Carbon isotope data have not been widely investigated in Cretaceous multi-taxa assemblages and studies have mainly focused on herbivore terrestrial taxa (e.g., [24–26]). We have analyzed δ^13^C values of sauropods, theropods, crocodyliforms and turtles as well as δ^13^C values of the bulk organic matter from the “Lo Hueco” sediments. Sauropod mean δ^13^C value is -10.5±0.8‰ and bulk organic matter mean δ^13^C value is -25.1±1.4‰ (Fig. 7). While we are aware that the low number of “Lo Hueco” sauropod tooth enamel samples may prevent us from attaining conclusive results, we still believe that it is worth discussing them in the context of other isotopic studies focused on herbivore dinosaurs. The “Lo Hueco” bulk organic matter mean δ^13^C value is almost identical to the estimated Late Cretaceous C_3_ vegetation mean δ^13^C value (-25.5‰), assuming a δ^13^C_atmCO2_ value of ∼-6‰ for the late Campanian-early Maastrichtian [57] and the modern fractionation value of ∼-19.5‰ between δ^13^C_atmCO2_ and C_3_ vegetation δ^13^C values [60]. This suggests a pure C_3_ environment for the “Lo Hueco” locality for that time period. Assuming that the “Lo Hueco” bulk organic matter mean δ^13^C value is representative of the δ^13^C value of the vegetation present in the area, then the fractionation between the “Lo Hueco” sauropod enamel and diet (Δ^13^C_enamel-diet_) would be ∼15‰. This offset lies between the one estimated for mammals and ostriches (12 to 14‰: [61–63, 66]) and the one estimated for sauropod dinosaurs and ostriches (∼16‰: [32, 65], respectively), and it is narrower when compared to the one estimated for ornithischian dinosaurs (i.e., 18‰: [25–26]). Stanton-Thomas and Carlson [24], Fricke and Pearson [25] and Fricke *et al*. [26] obtained higher δ^13^C values for hadrosaur and ceratopsian tooth enamel (-4‰ to -5.9‰) from different Late Cretaceous North American localities located in the vicinity of the Western Interior Seaway when compared to “Lo Hueco” sauropod δ^13^C values. Since bioapatite δ^13^C values of terrestrial vertebrates are ultimately controlled by δ^13^C values of diet giving rise to consistent Δ^13^C_enamel-diet_ values [63, 86], the difference between the fractionation values obtained in our study and those from Stanton-Thomas and Carlson [24], Fricke and Pearson [25] and Fricke *et al*. [26] might be likely associated to metabolic differences among taxonomic groups (sauropods *vs*. ceratopsians and hadrosaurs) (Fig. 8) [22–23, 46–48]. Bulk organic matter mean δ^13^C values showed by Fricke and Pearson [25] are ∼-24.0‰, just 1‰ higher than our bulk organic matter mean δ^13^C values, supporting differences in dinosaur metabolism in a scenario with similar isotopic values of vegetation (Fig. 7). Fricke and Pearson [25] pointed out that differences in the methane production in herbivore dinosaur stomachs during digestion along with different utilization of plant organic compounds (e.g., carbohydrates, proteins, lipids) and/or plant parts (e.g., leaves, seeds, wood) may also explain the differences observed in Δ^13^C_enamel-diet_. The larger ceratopsian and hadrosaur Δ^13^C_enamel-diet_ may be due to their ability to incorporate low quality, fibrous vegetation [25], whereas the smaller sauropod Δ^13^C_enamel-diet_ value may be indicative of consumption of more digestible food items (e.g., soft leaves), fact also supported by the dental morphology of “Lo Hueco” titanosaur sauropods (i.e., chisel-like teeth) more adapted to leave behind the least digestible tissues [98–99]. The 3‰ difference observed in Δ^13^C_enamel-diet_ values for ornithischians from Fricke and Pearson [25] and Fricke *et al*. [26] and sauropods from our study is statiscally significant (t = -4.481, p = 0.003), and therefore, even considering the variability yielded by dinosaur and bulk organic matter δ^13^C values, these two sets of data do not significantly overlap. Stanton-Thomas and Carlson [24] stated that higher δ^13^C values observed in hadrosaurs might be related to ingestion of vegetation subject to high salinity levels and/or consumption of gymnosperms (which show an enrichment of ∼1.1 to 2‰ compared to mean C_3_ δ^13^C values). “Lo Hueco” sauropods may have relied heavily on angiosperms as revealed by the palynological content of the “Lo Hueco” sediments showing pollen assemblages dominated by angiosperms (93%: [100]). Specifically, the palynological assemblage from “Lo Hueco” is dominated by freshwater palynomorphs, spores and pollen grains related to swamp or local wetland vegetation [100]. The preferential incorporation of angiosperms by “Lo Hueco” sauropods may also lie behind the difference observed between our Δ^13^C_enamel-diet_ value and the one estimated by Tütken [32]. Interestingly, in the compilation of sauropod δ^13^C values carried out by this author, *Ampelosaurus* showed the lowest δ^13^C values, and according to Knoll *et al*. [73], one of the “Lo Hueco” sauropod taxa remains close to this genus. These uncertainties regarding herbivore dinosaur isotopic paleoecology open up new lines of investigation dealing with the question of the type of dietary behavior and physiology of different genera giving rise to different Δ^13^C_enamel-diet_ offsets.

“Lo Hueco” theropod mean δ^13^C value is -10.7±0.8‰, statistically indistinguishable from the sauropod mean δ^13^C value (-10.5±0.8‰) (Fig. 4, S2 Dataset). To the best of our knowledge, there are no isotopic studies dealing with herbivore and carnivore dinosaur feeding paleoecology from the same locality. Isotopic studies on ancient vertebrate predator-prey systems have mainly focused on Quaternary mammals [64, 101–103] and to a lesser extent on Miocene mammals [104]. Clementz *et al*. [64] observed a δ^13^C offset between predators (wolves) and their prey (moose and elks) (Δ^13^C_carnivore-herbivore_) of ∼ -1.3‰. These authors argued that lower tooth enamel δ^13^C values for carnivores might be due to differences in their digestive physiology in comparison to herbivores. The lack of significant differences between “Lo Hueco” carnivore and herbivore dinosaur tooth enamel δ^13^C values (Δ^13^C_carnivore-herbivore_ = - 0.2‰, S2 Dataset) may be due to differences in metabolic and/or physiological mechanisms between dinosaurs and mammals, however a more plausible explanation is that titanosaur sauropods were not likely prey for theropods belonging to Dromaeosaurinae and Velociraptorinae subfamilies on account of body mass differences. Interestingly, the lack of significant differences in δ^13^C values between both theropod subfamilies (t = 0.516, p = 0.618), along with the similarity in their body masses and likely hunting strategies point to some degree of resource overlap. Also, the lack of significant differences in variance of δ^13^C values between these subfamilies of theropods (Levene test; F = 1.582, p = 0.240) suggests that none was more versatile than the other in resource utilization.

**Fig 9:**
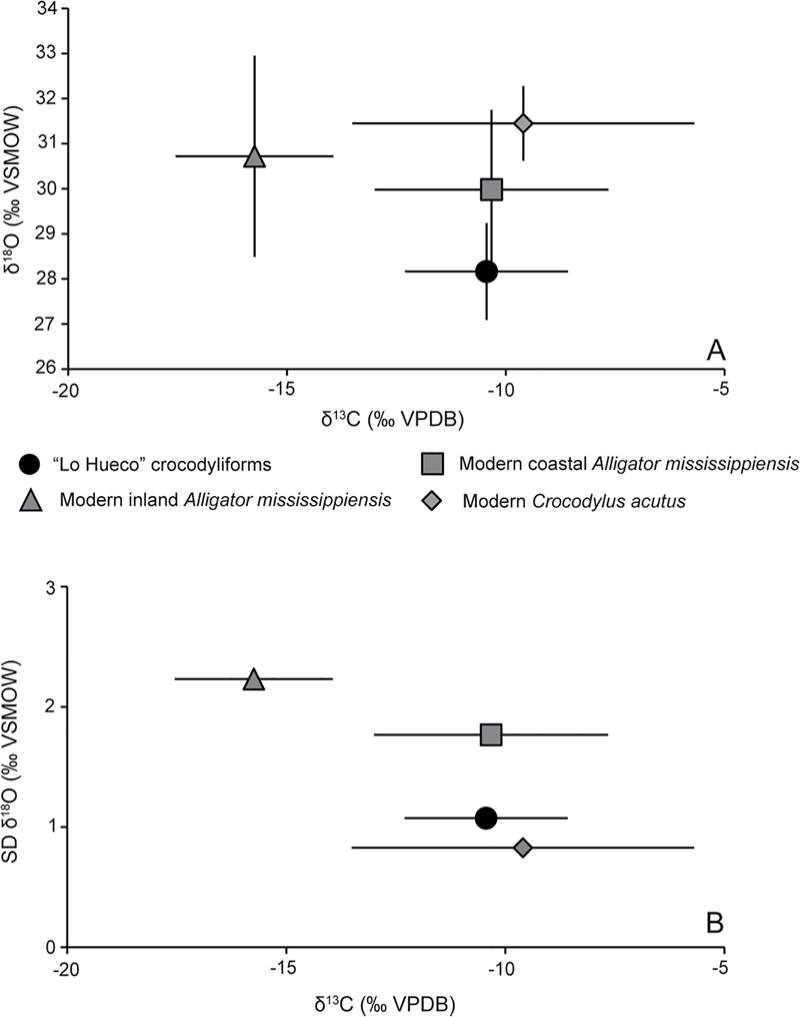
Comparison between isotopic values of “Lo Hueco” crocodyliforms and modern crocodilians. A) δ^18^O_CO3_ (‰ VSMOW) *vs* δ^13^C (‰ VPDB) mean ± 1 standard deviation (SD) values of the “Lo Hueco” crocodyliforms (black circle), and modern inland *Alligator mississippiensis* (grey triangle), coastal *Alligator mississippiensis* (grey square) and *Crocodylus acutus* (grey diamond) [107]. B) SD δ^18^O_CO3_ (‰ VSMOW) *vs* δ^13^C (‰ VPDB) values of the “Lo Hueco” crocodyliforms and modern crocodilians (symbols as in A).

In the case of aquatic ectotherms (crocodyliforms and turtles), δ^13^C values are a reflection of food oxidation (respiration) and ambient water (e.g., disolved inorganic carbon or DIC can constitute an additional source of carbon) [105–106]. “Lo Hueco” crocodyliforms belong to forms close to the genus *Allodaposuchus* [74]. Since it is not possible to determine physiological tolerance to salinity from the morphological standpoint in the basal Eusuchia from “Lo Hueco”, (J.L. Sanz and F. Ortega, personal communication), the information supplied by stable isotopes may shed light about salt tolerance, dietary behavior and habitat occupancy. We have compared the isotopic values of “Lo Hueco” crocodyliforms to those provided by Wheatley *et al*. [107] for Louisiana and Florida modern crocodylians. We are aware that extrapolation between extinct and extant organisms is not straightforward due to unknown physiological mechanisms in ancient taxa as well as different environmental conditions. However, we still believe that a comparison can be made since 1) the marine-freshwater isotopic threshold values are well established not only in modern studies, but also in the past, 2) the marine δ^18^O value has remained fairly constant throughout the geological time and 3) the Wheatley *et al*. [107]´s study considered modern crocodylians from Louisiana and Florida with a similar latitude to the one suggested for “Lo Hueco” (25–30°N for Louisiana and Florida *vs* 31°N for “Lo Hueco”). “Lo Hueco” crocodyliforms show a mean δ^13^C value, which is statistically indistinguishable from the value observed by Wheatley *et al*. [107] for Louisiana and Florida modern coastal *Alligator mississippiensis* (p = 0.999) and Florida modern coastal *Crocodylus acutus* (p = 0.868), while it is significantly different from Florida modern inland *A*. *mississippiensis* (p < 0.001), whereas “Lo Hueco” crocodyliforms show a significantly lower mean δ^18^O_CO3_ value, when compared to coastal and inland *A*. *mississippiensis* (p = 0.019 and p = 0.001, respectively) and coastal *C*. *acutus* (p < 0.001) (Fig. 9A). These results pose interesting issues concerning the salinity discrimination and osmoregulation capacity of the “Lo Hueco” eusuchian crocodyliforms. Jackson *et al*. [108] in a study of salinity tolerance and osmoregulation mechanisms by modern crocodilians argued that reptiles inhabiting marine and estuarine waters keep a constant plasma osmolality by behavioral modifications (avoiding drinking seawater) and/or morphological adaptation (salt-secreting glands and reduced integumental permeability). Crocodyliforms from “Lo Hueco” are under study and with the current information we cannot determine their physiological tolerance to salinity from a morphological standpoint (e.g., presence or absence of salt-secreting glands) (JL Sanz and F Ortega, personal communication, 2014). While waiting for these morphological studies, isotopic results suggest that the “Lo Hueco” crocodyliforms may have incorporated food items from brackish waters as shown by their δ^13^C values, whereas they avoided ingesting this water and consumed preferentially freshwater, as suggested by their δ^18^O_CO3_ values. Clementz and Koch [109] and Wheatley *et al*. [107] argued that animals incorporating marine food items and drinking seawater show a lower variability in their δ^18^O_CO3_ values. Even though “Lo Hueco” crocodyliforms likely drank freshwater, its δ^18^O_CO3_ variability (1.0‰) is not as high as that shown by either inland (2.2‰) and coastal (1.8‰) *A*. *mississippiensis*, and it is more similar to the one shown by saltwater tolerant *C*. *acutus* (0.8‰) (Fig. 9B). This might indicate that δ^18^O values of the water ingested by the “Lo Hueco” crocodyliforms may have remained homogeneous during the time window in which bioapatite mineralized (i.e., warmest months of the year).

“Lo Hueco” turtle mean δ^13^C and δ^18^O_CO3_ values are the lowest when compared to dinosaurs and crocodyliforms (Fig. 4) (although significant differences are only observed between turtles and dinosaur δ^18^O_CO3_ values, S2 Dataset). “Lo Hueco” turtles belong to two forms of Pleurodira, specifically to members of Bothremydidae [29, 75–76]. They are omnivore freshwater turtles, so low δ^13^C values may be indicative of a diet based on a mixture of aquatic and terrestrial C_3_ vegetation and/or invertebrates, whereas low δ^18^O values may indicate ingestion of water with an inland source. Unlike crocodyliforms, there are not isotopic studies dealing with bone carbonate of freshwater turtles from the subtropics and they focused on marine turtles [105–106]. As observed in the case of “Lo Hueco” crocodyliforms, “Lo Hueco” turtles show a low variability in their mean δ^18^O_CO3_ value, in spite of inhabiting freshwater environments, when compared to modern marine loggerhead turtles reported by Wheatley *et al*. [107] (0.3‰ *vs*. 0.6‰, for “Lo Hueco” and marine loggerhead turtles, respectively) pointing to a low variability of δ^18^O_CO3_ values and presumably of the δ^18^O values of the ingested water for the time of bioapatite mineralization (i.e., warmest months of the year).

## Conclusions

Isotopic analyses on the multi-taxa terrestrial vertebrate assemblage of the “Lo Hueco” locality (central eastern Spain) provides valuable information about climatic and environmental conditions existing in southeastern Iberia during the late Campanian-early Maastrichtian (Late Cretaceous). Seasonal thermal varibility has been inferred as the difference between temperature of the warmest months (TWMs), supplied by crocodyliforms and turtles (whose bioapatite mineralizes during the warm season) and mean annual temperature (MAT), provided by theropods (that record ingested water throughout the year). “Lo Hueco” ΔTWMs-MAT results do not point to a significant different seasonal thermal variability to that observed today. From the paleoecological standpoint, δ^13^C values of the “Lo Hueco” taxa point to consumption of pure C_3_ vegetation, fact that agrees well with bulk organic matter δ^13^C values from the “Lo Hueco” sediments. The estimated fractionation between sauropod enamel and diet (Δ^13^C_enamel-diet_) is ∼15‰, lower than other fractionation values calculated for sauropods (∼16‰) and ornithischians (∼18‰), and likely indicating differences in metabolic and/or physiological processes or different utilization of plant organic compounds and/or plant parts. Since “Lo Hueco” titanosaur sauropods may have not been likely prey for theropods belonging to Dromaeosaurinae and Velociraptorinae subfamilies on account of differences in their body mass, no conclusive information concerning dinosaurian predator-prey δ^13^C offset could be attained. Although “Lo Hueco” crocodyliform material remains under study and no paleoecological conclusions have been drawn from a morphological standpoint, isotopic results indicate that they may have incorporated food items from brackish waters as shown by their δ^13^C values, whereas they avoided ingesting saline water and consumed preferentially freshwater, as suggested by their δ^18^O_CO3_ values, when compared with isotopic values of modern crocodilians inhabiting subtropical regions. “Lo Hueco” turtles show the lowest δ^13^C and δ^18^O_CO3_ values of the vertebrate assemblage likely indicating a diet based on a mixture of aquatic and terrestrial C_3_ vegetation and/or invertebrates and ingestion of water with an inland source, a fact that agrees well with their taxonomic designation.

## Supporting Information

S1 Dataset Signature, level, sample, δ18OPO4 (‰ VSMOW), δ13C (‰ VPDB), δ18OCO3 (‰ VSMOW), and δ18OH2O (‰ VSMOW) values. (XLS)

S2 Dataset ANOVA and post-hoc tests for δ18OPO4, δ18OH2O, δ13C and δ18OCO3 values for the total dataset (TOTAL), and G1 and G2 levels. (XLS)

S3 Dataset Continent, country, station, latitude, longitude, altitude, year of record, mean annual temperature (MAT), temperature of the warmest months (July, August, September) (TWMs) and difference between TWMs and MAT (ΔTWMs-MAT). (XLS)

S4 Dataset A) ΔTWMs crocodyliforms-MATtheropods and ΔTWMs turtles-MATtheropods calculated using equations (4), (5) and (6) for the total dataset (TOTAL) and independently for levels G1. (XLS)

S5 Dataset Post-hoc Tukey p-values comparing pairs of ΔTWMs-MAT values for “Lo Hueco” locality, Late Cretaceous eastern Tethyan rudists [36, 38] and modern meterological stations from different continents (http://www-naweb.iaea.org/napc/ih/IHS_resources_isohis.html). (XLS)
